# Late right ventricular performance after mitral valve repair assessed by exercise echocardiography

**DOI:** 10.1007/s11748-018-0918-x

**Published:** 2018-04-05

**Authors:** Sigurdur Ragnarsson, Johan Sjögren, Martin Stagmo, Per Wierup, Shahab Nozohoor

**Affiliations:** 1 Department of Cardiothoracic Surgery, Skane University Hospital, Lund University, Getingevagen 4, 221 85 Lund, Sweden; 2 Department of Cardiology, Skane University Hospital, Lund University, Lund, Sweden

**Keywords:** Right ventricular function, Mitral valve repair, Exercise echocardiography

## Abstract

**Objectives:**

The aim of the study was to evaluate the right ventricular (RV) performance during exercise in patients who underwent mitral valve repair for chronic mitral valve insufficiency relative to healthy individuals and to assess exercise capacity using a semisupine ergometer.

**Methods:**

We studied 56 patients who underwent mitral valve repair for degenerative posterior mitral leaflet prolapse between 2005 and 2014 and a control group of 13 healthy individuals. Clinical data were collected prospectively, and echocardiographic measurements of RV function were obtained at rest and at peak exercise.

**Results:**

One-third of the study patients had RV systolic dysfunction as indicated by tricuspid annular plane excursion (TAPSE) at rest. Resting TAPSE was lower in the study group (16.7 ± 3.3 mm) than in the control group (24.4 ± 4.3 mm), *p* < 0.001. TAPSE increased in both groups during exercise and exercise was shown to have a significant main effect on TAPSE *F*(1, 52) = 80, *p* < 0.001. TAPSE increased more in the control group and an interaction was detected between the participant groups (study group vs. control group) and exercise, *F*(1, 52) = 24, *p* < 0.001. In the study group, Poor postoperative RV function was associated with preoperative left ventricular dilatation but was not correlated with impaired maximum exercise capacity.

**Conclusions:**

Despite the excellent clinical outcome during rest and exercise after mitral valve repair, our results suggest patients that have undergone mitral valve repair due to posterior leaflet prolapse have significantly reduced RV function at rest and during exercise compared to healthy controls at long-term follow-up, as measured by TAPSE.

## Introduction

Right ventricular (RV) dysfunction is a common finding in patients with chronic organic mitral insufficiency (MI) who are referred for surgery [1] and has been shown to be a negative prognostic marker following surgery [1, 2]. In the latest European guidelines [3], assessment of RV function and measurement of pulmonary artery systolic pressure (PASP) are recommended as a part of the preoperative evaluation in patients with chronic MI. RV dysfunction in preoperative chronic organic MI appears to be the result of a complex interaction between the remodeled and enlarged left ventricle (LV) in which interventricular septal function appears to be an important contributor [1], whereas the association between pulmonary hypertension and RV dysfunction is weaker [1]. Six months after mitral valve surgery, remaining RV dysfunction is common [4]. Poor postoperative RV function has been shown to be associated with both early and late mortality [2]. However, in multivariate analysis, it seems to be a surrogate for a poor preoperative clinical condition [2]. Data on long-term postoperative RV function at rest and especially during exercise are scarce, and the association between exercise capacity and RV dysfunction is not fully elucidated.

The evaluation of RV function is challenging due to the RV chamber’s complex geometry, the limited definition of the RV endocardial surface, and the marked load dependence of indices of RV function [5]. Measurement of tricuspid annular plane systolic excursion (TAPSE) is simple, not heavily dependent on optimal image quality, and reproducible [6]. It has high specificity and negative predictive value for detecting abnormal RV systolic function [7]. The contractile pattern of the RV changes following surgery with a relative shortening of the longitudinal contraction and relative increase in transverse shortening [8]; however, TAPSE has been shown to be an equally useful measure of RV function preoperatively and postoperatively in patients undergoing mitral valve surgery for degenerative disease with or without concomitant tricuspid valve surgery [9].

The aims of this study were to evaluate the RV function in patients who underwent isolated mitral valve repair for chronic organic MI due to posterior leaflet prolapse relative to healthy controls and to evaluate whether exercise capacity is associated with RV function.

## Methods

### Patient population

Between January 2005 and January 2014, 152 patients underwent primary repair of isolated posterior mitral leaflet (PML) prolapse due to degenerative disease. The exclusion criteria were: (1) residency outside of Skane County, (2) age > 80 years, (3) deceased during follow-up, (4) need of a translator, (5) chronic atrial fibrillation, (6) multiple repair techniques were employed, (7) severe annular calcification according to the operative report, (8) a rigid annuloplasty ring was used, (9) robotic-assisted surgery, (10) LV ejection fraction < 45% following surgery based on the most recent echocardiography, (11) severe aortic stenosis or severe aortic regurgitation based on the most recent echocardiography, (12) recurrent moderate or severe MI, and (13) reoperation due to recurrent MI.

Seventy-five patients met the inclusion criteria; of these, 43 underwent repair with resection techniques, and 32 underwent repair with artificial chordae. An invitation to participate was sent, and the patients were later contacted by telephone. Subsequently, 59 patients consented to participate; of these, 33 underwent leaflet resection, and 26 underwent repair with artificial chordae. One patient (artificial chordae) was not able to participate due to illness; one patient (resection) was not able to exercise due to rheumatic arthritis; and one patient exercised but was excluded from analysis because of digital storage failure. The remaining 56 patients had a mean cumulative follow-up interval from surgery to the exercise of 5.5 ± 3.2 years [median, 4.5 years; interquartile range (IQR) 2.8–8.0 years].

### Control group

Each of the 56 study patients was sex- and age-matched with an individual from the National Registry of those living in Skane County. A letter of invitation to participate as a control in the study was sent, and these individuals were subsequently contacted by telephone. The exclusions criteria were the same as for the study group in addition to known heart failure and ischemic heart disease. Fourteen individuals met the inclusion criteria and consented to participate. One patient could not exercise due to back pain. Thus, 13 individuals comprised the control group.

### Study protocol

The study protocol was approved by the Regional Ethical Review Board in Lund, Sweden (2014/784 and 2016/389). Prior to exercise echocardiography, the previous and current medical histories were assessed, and a physical examination was performed. All postoperative medical conditions, current medications, and the current NYHA class were recorded. The previous postoperative echocardiograms were reviewed for comparison. Functional capacity was assessed with the Duke Activity Status Index [10]. A 12-lead ECG was recorded to confirm the presence of sinus rhythm as well as to exclude conduction abnormalities and signs of myocardial ischemia. A TAPSE value of < 16 mm and/or an *S*′ value < 10 cm/s was defined as RV systolic dysfunction [6].

### Echocardiography

Resting transthoracic echocardiography study was conducted with the GE Vivid 7 ultrasound system (General Electric Healthcare, Boston, MA) with ECG trigger; a minimum of three loops were recorded. The semisupine exercise echocardiography test was performed on an eBike EL Ergometer (General Electric, Boston, MA). RV function was assessed using TAPSE and RV *S*′. TAPSE was recorded at the RV-free wall from a 2-dimensional-guided apical 4-chamber view; the M-mode cursor was placed through the tricuspid annulus, so that the annulus moved along the M-mode cursor. The total systolic displacement was measured from end diastole (beginning of QRS complex of the ECG) to the point of greatest contraction using the leading edge of the echoes. *S*′ was acquired using an apical 4-chamber window with a tissue Doppler mode region of interest that highlighted the RV-free wall. The pulsed Doppler sample volume was placed at the tricuspid level of the RV-free wall.

### Exercise echocardiography

The ergometer was adjusted, so that female participants started with 30 W resistance and male participants with 50 W resistance. The ergometer increased the resistance automatically by 10 W per minute. The participants were encouraged to exercise to their maximum capacity unless they experienced chest pain. When they reached their maximum capacity, the resistance was decreased by at least 30 W and the ergometer was rotated to the left to facilitate echocardiography. The participants were then asked to continue exercising for about a minute, while the echocardiography was performed. The blood pressure and heart rate were measured manually before exercise and every 3 min during exercise. Signs of arrhythmias and ischemia were monitored using continuous 3-lead ECG.

### Statistical analysis

Continuous variables that were distributed normally were expressed as the mean ± standard deviation (SD), whereas nonparametric continuous variables were expressed as the median and interquartile range (IQR). Student’s *t* test was used to evaluate normally distributed continuous variables, whereas the Mann–Whitney *U* test was used for nonparametric continuous variables. The repeated-measures ANOVA test was used to assess the effect of the participant group and exercise on TAPSE and *S*′. The chi-squared test was used for categorical variables, except when the expected frequency was below five, in which case Fisher’s exact test was used. A simple linear regression was used to assess the correlation between exercise capacity and RV function. Statistical analyses were performed and graphs plotted with the statistical software package SPSS (Version 22.0, IBM, Armonk, NY).

## Results

### Study participants

Preoperative patient characteristics and operative data are shown in Table 1. The demographics and clinical assessment of the study participants at the time of follow-up are shown in Table 2. The mean ages of the study group and control group were similar (*p* = 0.63) at the time of the study and the proportions of females in both groups were the same. The participants’ self-rated functional capacities, according to the Duke Activity Status Index, were similar in both groups (*p* = 0.26). The use of beta-blockers and use of vasodilators were more frequent in the study group than the control group.

**Table 1:** Preoperative patient characteristics and operative data

	*n* = 56
Preoperative characteristics of the study group	
Age at surgery (years)	60.0 ± 9.7
Female gender	17 (30%)
NYHA class III–IV	24 (43%)
LVEF < 50%	3 (5.4%)
PASP > 60 mmHg	17 (30%)
Logistic EuroSCORE	3.0 ± 1.6
Operative data	
Ring type	
Cosgrove-Edwards^®^ mitral annuloplasty system	3 (5.4%)
Duran Ancore^®^ band	46 (82%)
Physio II^®^ semirigid ring	7 (12.5%)
Affected scallop	
P1	3 (5.4%)
P2	55 (98%)
P3	8 (14.3%)
Prolapse of 2 or 3 scallops	9 (16%)
Type of repair	
Leaflet resection	32 (57%)
Artificial chordae	24 (43%)
Ring size (mm)	31.4 ± 2.7
ECC time (min)	110 ± 29
Cross-clamp time (min)	84 ± 23

Dichotomous variables are given as *n* (%); continuous variables are expressed as mean ± SD

*ECC* extracorporeal circulation, *EuroSCORE* European System for Cardiac Operative Risk Evaluation, *LVEF* left ventricular ejection fraction, *PASP* pulmonary artery systolic pressure

**Table 2:** Comparison of clinical assessment, heart rate, blood pressure, and measurements from exercise echocardiography between study and control groups

	Study group (*n* = 56)	Control group (*n* = 13)	*p* value
Clinical assessment			
Age at assessment (years)	65.5 ± 10.0	64.0 ± 9.7	0.63
Female gender	17 (30%)	4 (30%)	1.0
NYHA I/II/III/IV	46/9/1/0	12/1/0/0	
Short form assessment (Duke Activity Status Index)	52 ± 10	55 ± 4	0.26
BSA according to DuBois formula	1.95 ± 0.21	2.0 ± 0.18	0.47
Current medications			
Beta blocker	35 (63%)	3 (23%)	0.01
Calcium channel blocker	7 (13%)	0	0.33
ACE inhibitor or ARB	21 (38%)	1 (8%)	0.03
Resting heart rate and blood pressure			
Atrial fibrillation	0	0	
Resting heart rate (BPM)	75 ± 11	74 ± 11	0.74
Resting systolic BP (mmHg)	137 ± 15	138 ± 13	0.9
Measurements during exercise			
Maximum heart rate (BPM)	128 ± 20	129 ± 18	0.87
Maximum systolic BP (mmHg)	174 ± 31	181 ± 45	0.48
Duration (min)	9.1 ± 3.5	11.1 ± 4.1	0.33
Maximum effort (W)	133 ± 41	154 ± 47	0.12

Dichotomous variables are given as *n* (%); continuous variables are expressed as mean ± SD, unless they are nonparametric, in which case they are expressed as median (IQR)

*ACE* angiotensin converting enzyme inhibitor, *ARB* angiotensin II receptor blocker, *BP* blood pressure, *BSA* Body Surface Area, *NYHA* New York Heart Association

### Exercise capacity

The study group had a maximum exercise capacity of 133 ± 41 W, whereas the control group had a maximum exercise capacity of 154 ± 47 W (*p* = 0.12). The mean exercise duration was 9.1 ± 3.5 min and 11.1 ± 4.1 min in study group and control group, respectively (*p* = 0.33). The maximum heart rate was 128 ± 20 beats per minute in the study group and 129 ± 18 beats per minute in the control group (*p* = 0.9) (Table 2).

### Right ventricular function

**Fig. 1:**
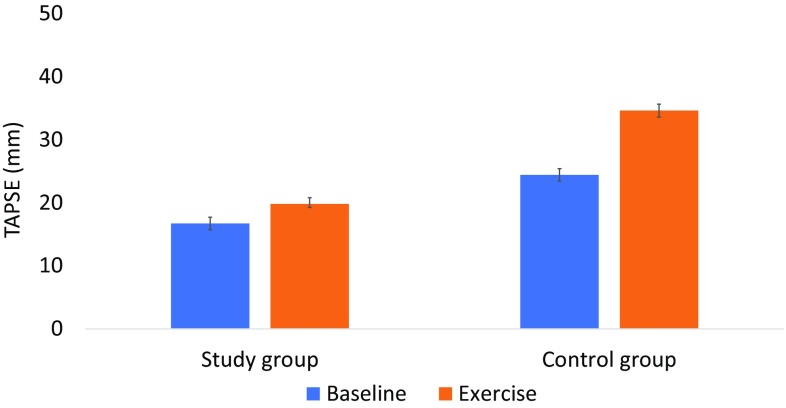
Tricuspid annular plane systolic excursion (TAPSE) during rest and peak exercise

**Fig. 2:**
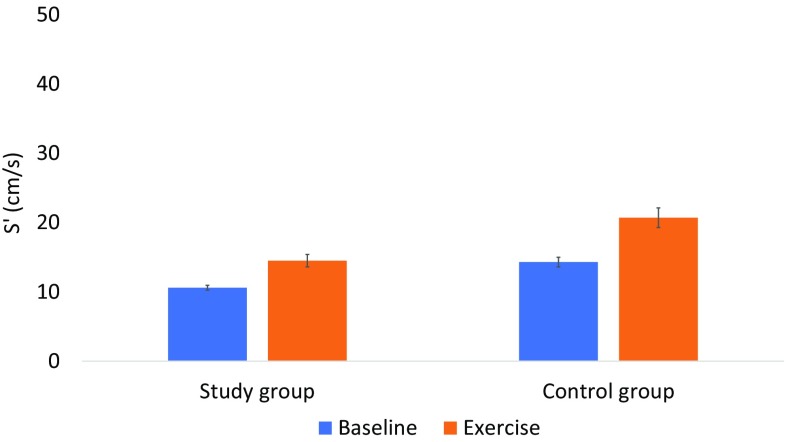
Tissue doppler-derived systolic velocity of the annulus (*S*′) during rest and peak exercise

The echocardiographic measures at rest and peak exercise are shown in Table 3. Resting TAPSE was lower in the study group (16.7 ± 3.3 mm) than in the control group (24.4 ± 4.3 mm), *p* < 0.001. Based on TAPSE assessed at rest, 33% (17/52) of the study group had RV dysfunction (TAPSE < 16 mm), whereas none of the control group had RV dysfunction. Figure 1 shows TAPSE measurements at rest and during peak exercise. The repeated-measures ANOVA test showed a significant main effect of exercise *F*(1, 52) = 80, *p* < 0.001. In addition, an interaction was detected between the participant groups (study group vs. control group) and exercise, *F*(1, 52) = 24, *p* < 0.001. In the study group, there was no correlation between baseline TAPSE and maximum exercise capacity (*R* = 0.064, *R*^2^ = 0.004) nor between TAPSE at peak exercise and maximum exercise capacity (*R* = 0.097, *R*^2^ = 0.009). Resting *S*′ was lower in the study group, 10.6 ± 2.6 cm/s, than in the control group, 14.3 ± 2.5 (*p* < 0.001). Based on *S*′ value, the study group, 44% (24/55) had RV dysfunction (*S*′ value < 10 cm/s), whereas none of the control group had RV dysfunction. As shown in Fig. 2, *S*′ increased in both groups during exercise. A significant main effect of exercise was detected *F*(1, 46) = 34, *p* < 0.001, and the interaction between the participant group and exercise *F*(1, 46) = 4.7, *p* = 0.036.

**Table 3:** Comparison of resting and exercise hemodynamics between study and control groups

Measure	*n*	Study group (*n* = 56)	*n*	Control group (*n* = 13)	*p* value
Baseline measures					
Any mitral insufficiency	56	10 (18%)	13	0	0.19
Mild		9 (16%)		0	
Moderate		0		0	
Severe		1 (2%)		0	
SAM	56	5 (9%)	13	0	1.0
Indexed LA volume (mL/m^2^)	45	35.7 ± 11.2	0		
LVEF (%)	51^a^	62.0 ± 6.0	6^a^	59.7 ± 3.1	0.29
LVIDd (mm)	53	45.5 ± 5.4	11	43.7 ± 6.5	0.31
LVPWd (mm)	53	12.1 ± 1.8	11	11.3 ± 1.1	0.17
Mean mitral valve gradient (mmHg)	52	3.0 ± 1.1	2	0.7 ± 0.1	0.005
*E*/*e*′	48	15.9 ± 4.3	11	7.1 ± 2.9	< 0.001
Peak aortic gradient (mmHg)	53	6.1 ± 2.9	12	9.4 ± 10.2	0.042
TRmaxPG (mmHg)	42	23.8 ± 5.5	3	22.1 ± 4.0	0.54
Interventricular septal movement (mm)	48	7.0 ± 1.3	10	8.9 ± 2.0	< 0.001
TAPSE (mm)	52	16.7 ± 3.3	12	24.4 ± 4.3	< 0.001
*S*′ (cm/s)	55	10.6 ± 2.6	12	14.3 ± 2.5	< 0.001
Peak exercise measures					
Any mitral insufficiency (grade 1–4)	56	11 (20%)	13	0	0.11
Mild		10 (18%)		0	
Moderate		0		0	
Severe		1 (2%)		0	
SAM	56	5 (9%)	13	0	1.0
Mean mitral gradient (mmHg)	51	10.1 ± 7.2	0		
Peak aortic gradient (mmHg)	46	11.1 ± 3.8	12	17.2 ± 13.9	0.01
TRmaxPG (mmHg)	29	38.8 ± 11.9	4	23.9 ± 9.4	0.03
TAPSE (mm)	40	19.8 ± 4.2	12	34.6 ± 3.8	< 0.001
*S*′ (cm/s)	33	14.5 ± 6.7	13	20.7 ± 5.1	0.005

Dichotomous variables are given as *n* (%); continuous variables are expressed as mean ± SD, unless they are nonparametric, in which case they are expressed as median (IQR)

*ACE* angiotensin converting enzyme, *ARB* angiotensin II receptor blocker, *BP* blood pressure, *BSA* body surface area according to DuBois formula, *E/e′* ratio of mitral peak velocity of early filling (*E*) to early diastolic mitral annular velocity (*e*′), *LVEF* left ventricular ejection fraction, *LVIDd* left ventricular internal diameter end diastole, *LVPWd* left ventricular posterior wall end diastole, *NYHA* New York Heart Association classification, *S′* Tissue doppler-derived systolic velocity of the annulus, *SAM* systolic anterior motion, *TAPSE* tricuspid annular plane systolic excursion, *TRmaxPG* tricuspid regurgitation maximum pulse gradient

^a^According to Simpson’s method, the remaining participants did not have sufficient image quality for the Simpson’s method, but all had normal ejection fractions based on visual judgment

### Left ventricular interdependence

Preoperative LV dilatation [left ventricular internal diameter end systole (LVIDs) ≥ 40 mm] was present in 34% of the patients. At follow-up, patients with preoperative LV dilatation had a mean TAPSE of 14.8 ± 2.4 mm at rest compared to 17.2 ± 3.4 mm in those with normal LV size (*p* = 0.037). However, during peak exercise, the difference in mean TAPSE did not remain significant. In the study group, 42 (75%) patients had tricuspid insufficiency permitting measurement of the peak gradient over the tricuspid valve (TRmaxPG). The TRmaxPG at rest was 24 ± 6 mmHg. Preoperatively, 17 patients (30%) had an estimated PASP ≥ 60 mmHg. No study patient had pulmonary hypertension during the exercise test. The TRmaxPG could be measured in three patients (8%) in the control group (mean 22 ± 4 mmHg). At peak exercise, the TRmaxPG remained unchanged in the control group; however, in the study group, it increased significantly from resting value of 24 ± 6–39 ± 12 mmHg (*p* < 0.001).

At follow-up, the left ventricular end-diastolic internal dimension (LVIDd) was 46 ± 5 mm in the study group and 44 ± 7 mm in the control group (*p* = 0.31). The left ventricular ejection fraction (LVEF) was 62 ± 6 in the study group and 60 ± 3 in the control group (*p* = 0.29). The mean left ventricular filling pressure, assessed with *E*/*e*′, was significantly higher in the study group than in the control group (15.9 ± 4.3 vs. 7.1 ± 2.9, respectively: *p* < 0.001).

## Discussion

In the present study, we could show that patients who underwent mitral valve repair due to posterior leaflet prolapse have significantly worse RV function than healthy controls at long-term follow-up. One-third of the study patients had RV systolic dysfunction as indicated by TAPSE. However, poor RV function was not associated with impaired maximum exercise capacity.

The previous studies have shown that RV dysfunction is common following cardiac surgery [11, 12]. Hyllén et al. showed that 6 months after mitral valve surgery for chronic degenerative mitral regurgitation, RV dysfunction, according to classical indices, was present in 61% [4]. Ye et al. [2] showed that, in patients operated for degenerative MI, TAPSE was related to both early and late death. The association did not remain significant after adjustment for preoperative risk factors, indicating that RV dysfunction is a marker of advanced disease before surgery [2]. Indeed, our study showed that patients with preoperative LV dilatation had lower TAPSE at rest at the time of exercise echocardiography compared to patients with normal preoperative LV size. In a large study by Desai et al. [9], in patients that did not have significant tricuspid regurgitation preoperatively, TAPSE was shown to decrease in the immediate postoperative period but increase again toward normal levels during the following years. The study measured echocardiographic parameters at rest. RV function in patients who underwent mitral valve repair has not previously been studied using exercise echocardiography. In this study, exercise echocardiography was used to evaluate RV function at rest and during exercise. Our data showed that exercise carried a significant main effect on both TAPSE and *S*′. In addition, we detected a significant interaction between the participant group and exercise in both TAPSE and *S*′, indicating that not only does the longitudinal contraction decrease following mitral valve surgery, but the contractile reserve also seems to be affected.

Despite having markedly lower than normal measures of longitudinal right ventricular function, based on TAPSE and *S*′, the patients in the study group had good exercise capacity with a near normal maximum workload; although the control group had a slightly higher exercise capacity, with no significant difference between the groups. The relationship between RV function and physical capacity following mitral valve surgery has not been previously reported. We did not find any correlation between TAPSE, whether evaluated at rest or peak exercise, and maximum workload. This may reflect an underestimation of RV function using the conventional longitudinal measures. Raina et al. showed that after cardiopulmonary bypass surgery and complete pericardiotomy, there was a marked reduction in longitudinal contraction and a relative increase in transverse parameters [8]. Hedman et al. performed a study of RV function and exercise performance in patients undergoing CABG [11]. The authors found that TAPSE significantly decreased following CABG despite improved exercise performance, suggesting that TAPSE may not be clinically significant following CABG surgery [11]. The observed reduction in RV function observed in that study might be due to geometrical rather than functional changes in the RV chamber as suggested in a previous study by Tamborini et al. [12].

### Limitations

The present study has inherent biases due to its nonrandomized design. The relatively small proportion of patients that were evaluated in the study among the initial cohort may limit the generalizability of the results. We did not obtain indices of RV radial function or RV volume measurements, because image quality was poor during exercise. The control group consisted of healthy individuals that had not undergone sternotomy and pericardiotomy. However, a control group of patients that have previously undergone sternotomy for other reasons is difficult to obtain and would introduce bias because of the respective operations that patients in such a group would have undergone. We could not evaluate all echocardiographic variables at peak exercise, because the image is of poorer quality; and it is more difficult to obtain good and reliable Doppler signals during peak exercise.

## Conclusion

The timing of mitral valve repair might be important to avoid persistent RV dysfunction at late follow-up after surgery. RV dysfunction due to longstanding degenerative MI is a marker of advanced disease supported in this study by the finding that preoperative LV dilatation was associated with lower TAPSE at follow-up, although impaired RV function did not correlate to poorer exercise capacity. Despite the excellent clinical outcome during rest and exercise after mitral valve repair, our results suggest patients that have undergone mitral valve repair due to posterior leaflet prolapse have significantly reduced RV function compared to healthy controls at long-term follow-up as measured by TAPSE and *S*′.
